# Factors Influencing Alzheimer's Anticipation in Midlife and Young Adulthood: American Perceptions of Aging in the 21^st^ Century [APA21], 2000

**DOI:** 10.1002/alz70858_099364

**Published:** 2025-12-25

**Authors:** Landon B Peeples, Lauren Chrzanowski, Benjamin Mast

**Affiliations:** ^1^ University of Louisville, Louisville, KY, USA; ^2^ University ​of Louisville, Louisville, KY, USA

## Abstract

**Background:**

Anticipation of Alzheimer's disease (AD) can impact health behaviors and psychological well‐being. Previous research has investigated influences of AD anticipation, highlighting the role of health concerns and psychological factors. However, research has focused on older adults to date, creating a gap in understanding anticipation of AD among individuals aged 18‐64.

**Method:**

Data from 1,749 participants aged 18‐64 were analyzed using the APA21 dataset, a nationally representative survey that explores attitudes and beliefs about aging among adults in the United States. Logistic regression was conducted with anticipation of AD as the dependent variable, classified as anticipating (0) or not anticipating (1.00). The sample was split into two age groups: 18‐39 (young adulthood) and 40‐64 (midlife). Predictors included self‐rated health, worry about memory loss, depression anticipation, education, gender, race, and religious importance. Model performance was evaluated using the classification table and fit indices (Cox & Snell and Nagelkerke

**Result:**

Of participants, 216 individuals (12.3%) anticipated developing AD. Poorer self‐rated health increased odds of anticipating AD (*B* = ‐0. 909, *p* = 0.026; Exp(*B*) = 0.403). Anticipating depression in later life was significantly associated (*B* = 0.667, *p* < 0.001; Exp(*B*) = 1.949). Worry about future memory loss was the strongest predictor (*B* = 1.784, *p* < 0.001; Exp(*B*) = 5.953), with those very worried about memory loss being almost six times as likely to anticipate AD. Age group (18‐39 vs. 40‐64) revealed a marginally significant negative association (*B* = ‐0.320, *p* = 0.051), suggesting that participants aged 40‐64 were slightly less likely to anticipate AD. Non‐significant predictors were education, gender, race, and religious importance. Although the model significantly improved prediction, it only explained about 7‐14% of variance in AD anticipation.

**Conclusion:**

Poorer self‐rated health, worry about memory loss, and anticipation of later life depression were significant predictors of AD anticipation, with individuals aged 40‐64 being slightly less likely to anticipate AD. These findings highlight the need for future research to evaluate if anticipation of AD influences risk reduction health behaviors. Additionally, it should aim to understand societal and individualized factors that shape AD anticipation and develop strategies to promote greater understanding of AD risk.

